# Testicular volume and Tanner stage: determinant factors for testicular torsion

**DOI:** 10.31744/einstein_journal/2022AO6605

**Published:** 2022-04-07

**Authors:** Aderivaldo Cabral Dias, Pedro Rincon Cintra da Cruz, Paulo Roberto Faria Ribeiro, Cassio Luis Zanettini Riccetto

**Affiliations:** 1 Hospital de Base do Distrito Federal Brasília DF Brazil Hospital de Base do Distrito Federal, Brasília, DF, Brazil.; 2 Hospital de Clínicas Universidade Estadual de Campinas Campinas SP Brazil Hospital de Clínicas, Universidade Estadual de Campinas, Campinas, SP, Brazil.

**Keywords:** Spermatic cord torsion, Ischemia, Testis/diagnostic imaging, Testis/growth & development, Ultrasonography, Sexual maturation

## Abstract

**Objective:**

To assess testicular volumes and sexual maturation in patients with testicular torsion.

**Methods:**

A retrospective analysis of consecutively treated patients with testicular torsion between 2016 and 2018. Age, pubic hair staging (Tanner), and by ultrasonography, volume of the unaffected testis (in cubic centimeters) were evaluated either immediately before surgery or at the first postoperative visit. Patients with previous testicular disease, such as cryptorchidism, or with no records of testicular volume were excluded. The analysis included descriptive statistics and Bayesian regression.

**Results:**

We treated 149 patients during the study period, and 141 (94.6%, median age 17.3 years) met the inclusion criteria. Median testicular volume was 13.0cm^3^ (interquartile range of 10.5-15.2), with similar right and left volumes (12.9cm^3^*versus* 13.3cm^3^; p=0.94). Sixty-five (46.1%) patients were Tanner stage IV, 17 (12.1%) stage III, and 59 (41.8%) stage V.

**Conclusion:**

In this study, we were able to estimate volumes of testicular torsion, which aggregated around late puberty values (13.0cm^3^ for the whole dataset, 12.2cm^3^ for patients <25 years), suggesting that testicular hypermobility, due to congenital anatomical abnormalities, remains quiescent until the organ reaches a critical volume, after which torsion becomes possible. These findings provide a tentative explanation for the disease’s age distribution.

## INTRODUCTION

Intravaginal testicular torsion (testicular torsion) occurs when the testis rotates on its axis inside the tunica vaginalis, which twists the spermatic cord and strangulates its vessels, hindering testicular blood flow. These events often cause testicular hemorrhagic necrosis should prompt treatment not be provided. Indeed, testicular torsion still is a common cause of organ loss,^(1)^ especially in emerging countries.^(2,3)^ The occurrence of testicular torsion requires testicular hypermobility due to anatomical abnormalities, such as bell clapper anomaly or an elongated mesorchium.^(4-6)^ These anomalies, however, are congenital, and testicular torsion mostly affects boys in late puberty,^(7-11)^ suggesting pubertal growth is involved in the pathogenesis of the disease. Also, testicular torsion is rare after the third decade of life,^(12)^ indicating that adult testicular volumes *per se* do not increase the probability of the disease. These arguments lead us to postulate that, in order to rotate, the testis must reach some critical volume, and, given the age distribution of testicular torsion patients, this volume should approximate the one attained at the end of puberty.^(13,14)^

We were, however, unable to find reports of testicular volumes in torsion. Thus, the goal of our study was to estimate these volumes, and correlate them with each patient’s stage of sexual maturation. In this endeavor we took advantage of the fact that our unit’s data repository contains not only testicular ultrasound, but also anthropometric and sexual development data.

## OBJECTIVE

To estimate contralateral testicular volume, and correlate it with Tanner’s sexual maturation stages in patients presenting with testicular torsion.

## METHODS

### Patient identification and data retrieval

After approval by the Institutional Board Review (CAAE: 45229515.6.0000.5553, protocols 1.076.823 and 3.947.222), informed consent was waived, we retrospectively identified from our unit’s data repository all consecutive patients surgically treated for testicular torsion, at the *Secretaria de Estado de Saúde do Distrito Federal*, between January 2016 and January 2018, and accessed their medical records for variable retrieval. We excluded from analysis, patients with a history of cryptorchidism, testicular or epididymal infections, previous testicular surgery or torsion, as well as those whose records lacked testicular measurements.

Intravaginal testicular torsion causes (often severe) swelling, and that the affected organ’s best substitute is the contralateral testicle, we computed contralateral testicular volumes in cubic centimeters, using the formula (length x width x depth x 0.52) from ultrasound measurements. These were made either immediately before surgery, with a 10MHz linear transducer (Sonosite, Fujifilm, Japan); or at the first postoperative visit, with a 10 to 15MHz linear transducer (Xario, Toshiba, Japan), and recorded as a continuous variable, whereas the occasion of measurement (before or after surgery) was recorded as a categorical variable. Body weight (in kilograms) and height (in centimeters) were retrieved from measurements taken at the first postoperative visit, with the patient barefoot and lightly clothed, by means of a calibrated scale with a built-in stadiometer (Filizola, São Paulo; sensitivity of 100g and 0.5cm, respectively), and also recorded as continuous variables. We also included, as a continuous variable, the number of days between surgery and first postoperative visit; and, as a categorical variable, and which testis was measured (right or left). Standard reference figures^(15)^ were used to classify each patient’s pubertal developmental stage according to their pubic hair (Tanner PH) distribution. Tanner PH stage was assessed at the first postoperative visit and recorded as an ordinal variable.

### Statistical analysis

Continuous variables were described by their medians and interquartile ranges (IQR), and frequencies were used to describe categorical and ordinal variables. Welch’s two-sample *t* tests were used to compare normally distributed continuous variables, and we used Kruskal-Wallis test to compare non-normally distributes variables. Normalcy of distribution was assessed with the Anderson-Darling test.

Bayesian linear regression models were employed to assess the distribution of testicular volumes in torsion, in the subset of patients with ongoing pubertal development, *i.e*. aged <25 years, using as explanatory variables age, and Tanner PH stage. Since these variables were highly correlated (Spearman’s p=0.86), separate models were implemented for each predictor.

In customary notation, the models were according to equations 1 and 2.


$${\text{ volume }}_{i}∼\text{ Normal }\left(\beta_{0}+\setminus \beta_{\text{age }}\times \left({\text{ age }}_{i}-\mathrm{mean}(\text{ age }),\tau \right)\right)$$ Equation 1

$$\text{ volume }{}_{i}∼\text{ Normal }\left(\beta_{0}+\setminus \beta_{\text{Tanner }}\times \left({\text{ Tanner }}_{i}-1\right),\tau \right))$$ Equation 2

Normal (0.5) prior distributions were used for the β_age_, β_Tanner_ and β_0_ parameters, with the latter truncated at the [0.5, 40] interval, and Gamma (0.001, 0.001) prior was used for the precision parameter τ. Models were written in the JAGS language using the rjags package^(16)^ as its interface to the R language.^(17)^ Posterior probability distributions were summarized by their means, and 95% highest density posterior intervals (HDPI).

## RESULTS

### Data retrieval

We treated 149 patients for testicular torsion in the study period, and eight were excluded from the study: one patient had a history of mumps orchitis, three had been treated for cryptorchidism, and four patients had a previous testicular torsion. All 141 (94.6%) remaining patients had recorded values of anthropometric and testicular measurements, as well as Tanner PH classifications, and were included in our analysis.

### Age, testicular volume, and measured side

**Figure 1:**
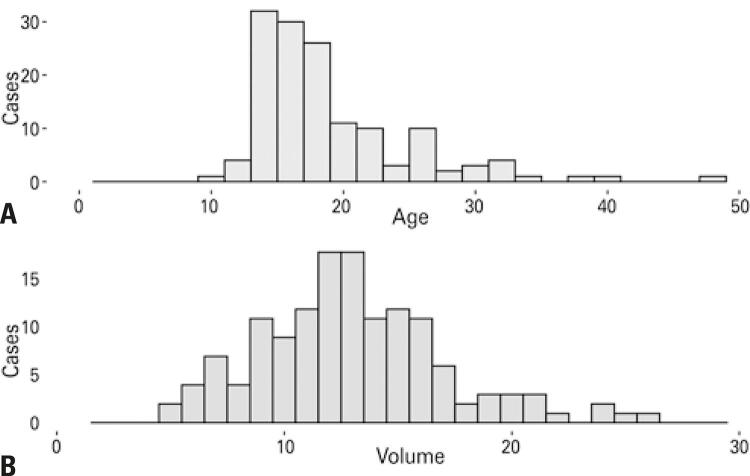
Distribution of patient’s age and testicular volumes. (A) Histogram of the distribution of patient’s age in years; (B) Histogram of the distribution of testicular volumes in cubic centimeters

Patients’ age and testicular volume were not normally distributed (p<0.001 and p=0.02, respectively; Anderson-Darling’s test; Figure 1; Table 1).

**Table 1:** Overall study population characteristics

Variable	Median (IQR)
Age, years	17.3 (14.7-21.3)
Volume, cm^3^	13 (10.5-15.2)
Height, cm	169.4 (166.3-173.0)
Weight, kg	62.5 (56.0-70.0)
Right side, n (%)	89/141 (63.1)
Tanner PH, n (%)	
III	17/141 (12.1)
IV	65/141 (46.1)
V	59/141 (41.8)

Results expressed as median (interquartile range) or n/total n (%).

**Figure 2:**
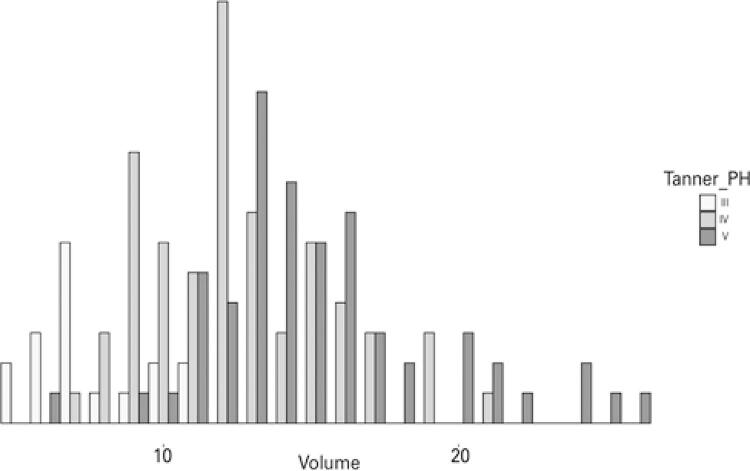
Histogram of the distribution of testicular volumes, in cubic centimeters, by Tanner’s pubic hair maturation stages (Tanner PH)

However, testicular volumes for patients aged <25 years were normally distributed (p=0.30; Anderson-Darling). Median age was 17.3 years (IQR 14.7-21.3). Most testicular volumes were measured at first examination (110/141; 78%), and they ranged from 5.0cm^3^-26.5cm^3^, with median of 13.0cm^3^ (IQR 10.5-15.2; Figure 2). Testicular volumes were similar regarding side (median 12.9cm^3^*versus* 13.3cm^3^ for right and left organs; p=0.94; Kruskal-Wallis) and occasion of measurement: volumes at first examination were similar to those measured at the first postoperative visit, which occurred at median 17 days (IQR 15-20) after surgery (mean 13.0cm^3^*versus* 13.4cm^3^ at the first return visit; p=0.43; Kruskal-Wallis).

### Tanner’s pubic hair maturation stage

Most patients were classified as stage IV (65/141, 46.1%), and none were classified as stage I or II. The first author was responsible for two-thirds of Tanner PH stage assessments (92; 65.3%), whereas the second author evaluated 29 (20.6%) and the third author 20 (14.2%) patients. The distribution of testicular volumes according to Tanner stage is shown in table 2, along with other characteristics of our study population, and is graphically displayed in figure 2.

**Table 2:** Distribution of variables according to Tanner pubic hair maturation stage

Variable	Tanner PH		

	III (n=17)	IV (n=65)	V (n=59)
Age, years	13.6 (13.1-13.8)	15.6 (14.5-17.3)	22.3 (19.5-26.8)
Volume, cm^3^	7.2 (6.0-8.7)	12.0 (10.2-14.8)	14.4 (12.8-16.5)
Height, cm	165.7 (162.0-167.3)	168.2 (166.1-170.0)	173.0 (170.0-176.5)
Weight, kg	60.0 (51.0-62.8)	57.0 (55.0-62.5)	71.0 (64.5-72.8)
Right side, n (%)	12 (70.6)	42 (64.6)	35 (59.3)

Results expressed as median (interquartile range) or n (%).

### Bayesian linear regression

A total of 117 patients (117/141; 83%) were aged <25 years and were included in this analysis. After examination of prior predictive distributions and convergence diagnostics, we generated 300 thousand Markov chain Monte Carlo posterior distribution samples for each model.

Posterior distribution mean testicular volumes were 12.2cm^3^ (HDPI 11.6-12.8). Mean volumes either increased or decreased by a mean of 0.51cm^3^ (HDPI 0.34-0.74) for each year of age either added to or subtracted from the age of 16.7 years (the calculated mean for this subset). Similarly, mean volumes at Tanner PH stage IV were 11.8cm^3^ (HDPI 11.2-13.4), decreasing or increasing by a mean of 2.8cm^3^ (HDPI 1.9-3.7), for stages III or V, respectively.

## DISCUSSION

In this case series, which included 141 testicular torsion patients, we found the median volume of non-affected testis was 13.0cm^3^, and most patients were advanced in their sexual maturation (none were less than Tanner P stage III). These findings were compatible with our conjecture that testicular torsion only occurs when the organ reaches a critical volume.

More than a century ago, Lauenstein^(18)^ noticed testicular torsion mostly affected young men and boys in late puberty, a finding that has since then been consistently replicated.^(2,3,7,19)^ Despite this possible connection between the occurrence of testicular torsion and pubertal testicular growth, research into this aspect of the disease pathogenesis has been limited to the description of congenital anatomical abnormalities associated with testicular hypermobility, such as the bell clapper anomaly, and testicular-epidydimal junctional disorders.^(4,5)^

The bell clapper anomaly consists of a high insertion of the tunica vaginalis associated with the lack of fixation of the testis to the scrotal wall, whereby the final segment of the spermatic cord is situated within the tunica cavity, so that the cord can twist *in tandem* with the testis and epididymis. This anomaly, estimated to occur in 12% of male population, has been reported in the majority of testicular torsion cases.^(4,5)^ Another anatomical abnormality less often associated with testicular torsion is a wide separation between testis and epididymis,^(5,6)^ enabling torsion to occur between these structures, *i.e*., intramesorchially.

**Figure 3:**
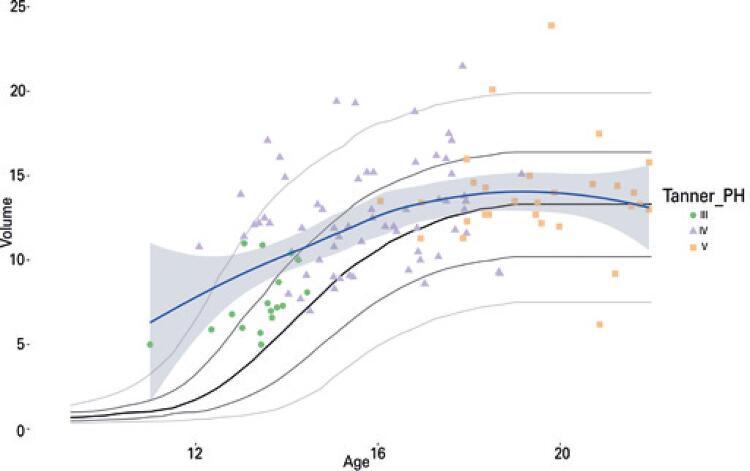
Scatterplot of testicular volumes in cubic centimeters (y-axis) by patients’ ages in years (x-axis) according to Tanner’s pubic hair maturation stages, with locally estimated smoothing and 95% confidence intervals (blue line and shadowed region), overlaid by Joustra’s. Ultrasound testicular growth reference curves (thick curve, median thin curves, from down upward: P0.02, P0.16, P0.84, P0.98)

Source: Joustra SD, van der Plas EM, Goede J, Oostdijk W, Delemarre-van de Waal HA, Hack WW, et al. New reference charts for testicular volume in Dutch children and adolescents allow the calculation of standard deviation scores. Acta Paediatr. 2015;104(6):e271-8.^(13)^

Yet, the mere existence of these congenital anomalies cannot explain why the disease overwhelmingly affects individuals in late puberty; were these anomalies the only anatomical predisposing factor for testicular torsion, one should expect torsion to occur in younger population strata. In fact, one can fathom that the full development of these anomalies requires the organ to reach a certain volume - approximately 13cm^3^, according to our study — which creates a spacious tunica cavity, with a large serosal low friction surface upon which the organ can slide, as well as a spermatic cord of sufficient length-to-width ratio to enable it to twist. This also suggests that other risk factors for the disease, *e.g*. exercise, minor trauma and low room temperature,^(19,20)^ would only precipitate torsion in the presence of such favorable anatomic configuration, which should occur earlier in early maturing individuals and later, in late maturators (Figure 3).

Differences in the degree of hypermobility of the organ, dependent upon the particular anatomical arrangement of the elements within the tunica cavity, *e.g*. the level of insertion of the tunica vaginalis, determining the length of the intravaginal spermatic cord segment; as well as of individual patterns of cremasteric activity (the postulated mechanism for the increase in the risk of torsion, with colder temperatures), and external mobilization (exercise, minor trauma), would account for additional variation of the likelihood of a torsion event, for which an entropic argument can be put forward: since configurations other than the normal one are possible in the hypermobile testis, the likelihood that the organ moves into another given position should be proportional to the probability of said position. Thus, as the probability of an alternative testicular position overcomes the probability of the normal position, torsion becomes more likely. This pathogenic model also implies the rarity of testicular torsion in older individuals results from a harvesting effect: as the occurrence of the disease in pubertal boys and young men depletes the population’s susceptible pool, the fraction of susceptible patients in older population strata becomes progressively smaller.

This progressive likelihood of acute testicular torsion may be clinically heralded by intermittent testicular torsion, characterized by spontaneously resolving hemiscrotal pain episodes with intervening asymptomatic periods. Intermittent torsion precedes acute testicular torsion in 30% to 50% of patients,^(21-23)^ and its identification and treatment has led to high organ salvage rates. Already in 1976, Williamson^(24)^reported salvaging the testis of all his 21 patients diagnosed with this condition. Similarly, American urologists^(21)^ reported a 100% salvage rate in patients electively treated for the disease, compared to a 47% salvage rate in those operated in the emergency setting, and investigators from Nigeria^(25)^ achieved an 88% salvage rate by pre-emptively treating 22 (out of 34) patients diagnosed with intermittent torsion.

The physical sign most consistently observed in patients with intermittent torsion is the horizontal lie of the testis when the patient is standing.^(26,27)^ This sign is strongly associated with the bell clapper anomaly,^(24,26,28)^ is more often observed during asymptomatic periods,^(27)^and could be used to screen susceptible patients. In this sense, a promising development has been recently reported by Tokuda et al.,^(29)^ who reported a hyperintense signal between the epididymis and tunica vaginalis (the “split sign”) on magnetic resonance T2-weighted images, which predicted the bell clapper anomaly in five out of six patients. This study, however, examined only acute testicular torsion cases, and these findings require external validation to assess its real-world applicability – especially in patients that are not in the acute phase of the disease.

Given the dismal testicular salvage rates still seen throughout the world, the time has come for the urology community to consider the prevention of acute testicular torsion. Hypothetically, patients in which sexual maturation is compatible with the disease, and with physical signs indicative of testicular hypermobility, would undergo counseling – or imaging studies, should they prove to be useful – and, in carefully selected situations, be offered pre-emptive orchidopexy. We are fully aware such approach would require evidence that still does not exist: a prospective randomized trial, for instance, which should be subjected to rigorous ethical scrutiny, in which perioperative (anesthetic, infectious) risks to the treated population and financial costs should be fully taken into account.

This study has many limitations, and we reckon measurement error to be the main one, with at least three possible sources. Firstly, we could not independently verify testicular measurements from actual ultrasound images, since we only had access to ultrasound reports from the on-call radiologist. Secondly, some patients might have had intermittent torsion before the acute event, so that our measurements, at or following the acute event, could have been biased towards larger volumes. Thirdly, due to testicular swelling after torsion, we had to use data from the non-affected organ – as we have mentioned in the methods section. Although we attempted to exclude patients with previous testicular disease, it is possible – quite likely, actually, as the smallest testis in the dataset belonged to a Tanner stage V patient – that some patients had undetected contralateral disease, thus generating bias towards smaller testicular volumes.

In addition, we cannot fail to mention that some patients did not neatly fit into Tanner’s PH maturation stages figures, and might have been misclassified regarding the sexual maturation stage. We also acknowledge that the 25 year-old limit we set for our regression analysis is, however reasonable, arbitrary. Lastly, since our database only contains surgically treated patients, our results are subject to selection bias, since it is possible that some testicular torsion patients, first examined by us, opted to be treated elsewhere (in private institutions), or even refused surgical treatment, and that the variables’ values from these patients could have changed our estimates.

## CONCLUSION

In this study, the median testicular volume of the non-affected organ in acute intravaginal testicular torsion patients was 13.0cm^3^, and that no patient had a testicular volume less than 5.0cm^3^. Also, most patients were advanced regarding their sexual maturation status (Tanner PH stage IV), and that no patient was classified as stage I or II. These findings not only better define the population at-risk for testicular torsion, but also lend support to the conjecture that the congenital anatomical anomalies associated with the disease remains quiescent, until the organ reaches or surpasses some critical volume, after which the testis becomes hypermobile, and torsion, possible.
